# Statistical Modeling and Analysis of Laser-Evoked Potentials of Electrocorticogram Recordings from Awake Humans

**DOI:** 10.1155/2007/10479

**Published:** 2007-08-06

**Authors:** Zhe Chen, Shinji Ohara, Jianting Cao, François Vialatte, Fred A. Lenz, Andrzej Cichocki

**Affiliations:** ^1^Laboratory for Advanced Brain Signal Processing, RIKEN Brain Science Institute, Wako-shi 351-0198, Saitama, Japan; ^2^Neuroscience Statistics Research Lab, Massachusetts General Hospital, Harvard Medical School, Boston, MA 02114, USA; ^3^Department of Neurosurgery, Johns Hopkins Hospital, Baltimore, MD 21287-7274, USA; ^4^Department of Neurosurgery, Kyoto Kizugawa Hospital 26-1, Nishi-Rokutan, Hirakawa Joyo 610-0101, Kyoto, Japan; ^5^Department of Electronics and Information Engineering, Saitama Institute of Technology, Fukaya-shi 369-0293, Saitama, Japan

## Abstract

This article is devoted to statistical modeling and analysis of electrocorticogram (ECoG) signals induced by painful cutaneous laser stimuli, which were recorded from implanted electrodes in awake humans. Specifically, with statistical tools of factor analysis and independent component analysis, the pain-induced laser-evoked potentials (LEPs) were extracted and investigated under different controlled conditions. With the help of wavelet analysis, quantitative and qualitative analyses were conducted regarding the LEPs' attributes of power, amplitude, and latency, in both averaging and single-trial experiments. Statistical hypothesis tests were also applied in various experimental setups. Experimental results reported herein also confirm previous findings in the neurophysiology literature. In addition, single-trial analysis has also revealed many new observations that might be interesting to the neuroscientists or clinical neurophysiologists. These promising results show convincing validation that advanced signal processing and statistical analysis may open new avenues for future studies of such ECoG or other relevant biomedical recordings.

## 1. INTRODUCTION

Pain is an essential function for the organism to enable immediate awareness of actual or threatening injury for further adopting a self-protective behavior. Roughly speaking, pain is a complex and subjective experience in the brain; it involves sensory, affective, cognitive, and motivational components and is associated with autonomous activity, nocifensive reflexes and reactions. In clinical practice, neurophysiological evaluation of pain in humans has been an important subject of research in the last decade (Bromm and Lorenz [1]).

In the literature, there are many approaches for monitoring and measuring the pain-related brain activities, including electroencephalogram (EEG), magnetoencephalogram (MEG), and fMRI. In particular, the electrocorticogram (ECoG) records directly the cortical (electrical) activities from subdural electrode grids that are implanted in the human subjects for collecting information for surgical treatments of medically intractable epilepsy (i.e., patients in the hospital upon approval). As an invasive recording tool, ECoG offers some superior features that are unavailable for EEG or MEG recordings. Specifically, unlike EEG that measures the electrical potentials recorded from the scalp, ECoG directly records the potentials from the cortical surface, thereby having a higher signal-to-noise ratio (SNR) and higher spatial resolution (because of closer electrode spacing). Consequently, activities in beta or gamma bands are better recorded in ECoG due to less spatial summation and phase cancelation (or high-cut filter effect) than in scalp EEG recordings.

Since the energy of the infrared laser can be used to produce a brief thermal stimulus applied to the skin such as to selectively activate the skin nociceptor, the recordings of brain responses to short laser pulses (the so-called laser-evoked potentials, or LEPs) have increasingly become a useful method for evaluating the function of central nociceptive pathways. The roles of LEPs for detecting abnormalities in patients have been noted (García-Larrea et al. [2]). Generally, there are two or three major peaks in the pain-evoked LEPs, which may be generated in multiple regions. In the literature, most research efforts focused on two peaks of the LEPs, the so-called N2 and P2, which correspond to the vertex negative-positive complex^1^ . The timing when the peak of the LEP appears is referred to the latency of LEPs. Typically, N2 was found around 150–400 milliseconds, and P2 was found around 230–500 milliseconds, depending on the laser pulse duration and intensity, as well as the stimulus site or area (Bromm and Lorenz [1]). The difference in latency is essentially related to the response differences in peripheral conduction distance. Specifically, LEP reflects an integrative cortical response to the painful laser stimuli rather than a simple reaction of the sensory cortex to it; thus, in the healthy subject the amplitude of cortical LEPs correlates with the subjective sensation of pain, rather than with the physical stimulus intensity (García-Larrea et al. [2]). For instance, paying attention to the laser stimulus simultaneously increases the subjective pain sensation and the LEP amplitude, both of which decrease in turn when the subject is distracted from the stimulus (García-Larrea et al. [3]). In addition to the amplitude, the latencies of the LEPs are often important for the neurophysiological evaluation of pain (Bromm and Lorenz [1]). In a later section, we will analyze the amplitudes and latencies of LEP components N2 and P2 in detail. As suggested in the literature, the negative component (N2) seems to be induced mainly by the activation in the bilateral operculoinsular cortices and contralateral primary somatosensory cortex (SI) (e.g., Tarkka and Treede [4], Iannetti [5]), and the positive component (P2) is mainly generated by the cingulate gyrus (e.g., Tarkka and Treede [4], Lenz et al. [6], Iannetti et al. [5]). However, it should also be noted that both N2 and P2 could be recorded and observed at multiple cortical regions simultaneously (e.g., Ohara et al. [7–9]); therefore, although there may be some evidence that one LEP is more related to a particular region than the other, a full understanding of their underlying mechanisms remains unclear.

In the previous studies (Ohara et al. [8]) of the ECoG recordings from the awake humans, it was found that attention to painful cutaneous laser stimuli enhances pain-related LEPs in cortical regions receiving nociceptive input, typically at multiple cortical sites (Ohara et al. [9]). Specifically, it was observed that at primary somatosensory (SI), parasylvian (PS), and medial frontal (MF: anterior cingulate and supplementary motor area) cortex areas, the amplitudes of the negative (N2^∗^) and positive (P2^∗^^∗^) LEP components^2^ were enhanced by attention to (counting stimuli), in comparison with distraction from the stimuli (reading for comprehension). It was suggested therein that attention controls both early (N2^∗^) and late (P2^∗^^∗^) pain-related input to SI (and other) cortical regions, while the late positive deflections (that follow the P2^∗^^∗^ peak) are specifically related to attention. It was also reported in other independent EEG studies (e.g., Legrain et al. [10, 11]) that LEPs can be modulated by selective spatial attention. In [7], Ohara et al. observed that attention to painful stimuli leads to enhanced event-related desynchronization (ERD) in cortical regions receiving input from nociceptors, and the alpha ERD is more widespread and more intense during attention to the laser than distraction from the stimuli. This was also consistent with the observations from other studies using EEG or MEG recordings (Mouraux et al. [12], Ploner et al. [13]).

In recent years, many statistical tools, such as principal component analysis (PCA), independence component analysis (ICA), parallel factor analysis (PARAFAC), common spatial subspace decomposition (CSSD), statistical wavelet thresholding (SWT), and Kalman filtering, have been used for analyzing biological or biomedical data, including EEG, MEG, and fMRI (e.g., Lee et al. [14], Cao et al. [15, 16], Makeig et al. [17], Anemüller et al. [18], Miwakeichi et al. [19], Browne and Cutmore [20], Wang [21], Galka [22], Cichocki [23, 24]). The common goal of these mathematical tools is to discover the hidden components underlying the data and extract the markers for characterizing specific events (e.g., event-related potentials). In addition, combing ICA or other statistical tools with advanced time-frequency analysis methods has also been advocated in cognitive neuroscience and neuroimaging (e.g., Makeig et al. [25], Mørup et al. [26]).

In this paper, we conduct both quantitative and qualitative analyses of ECoG data induced by pain stimuli controlled by a laser pulse. The investigation is focused on two selected human subjects under several different controlled stimuli conditions: attention, distraction, as well as under different laser intensity levels. Statistical analysis was conducted for both averaging trials and single trials. The averaging-trial study attempts to find out the dominant and common components (especially LEPs) by averaging all trials (of one subject) under the same conditions. In contrast, the single-trial study aims to search for instantaneous brain waves and to analyze the corresponding LEP properties (such as the amplitude and latency). The signal-trial analysis is important because the spontaneous brain activities that are regarded as “noise” are often diminished by averaging. We believe that the results obtained from the single trials, if analyzed appropriately, often offer extra information that is unavailable in the averaging-trial study (e.g., Makeig et al. [25]).

To achieve our goal, we select proper processing procedures and mathematical tools, including factor analysis (FA) and ICA, to the experimental recordings. This builds on the assumption that within a short timescale the ECoG recordings are approximated by an instantaneous linear generative model that is corrupted by additive noise. The LEPs of interest and other instantaneous brain activities are assumed to be mutually independent. To blindly separate the sources of interest (i.e., LEPs), we first resort on a dimensionality reduction procedure followed by an efficient and robust ICA estimation method. In addition, with eigenvalue decomposition, an energy ratio threshold is defined to reject nonsignificant components, which are regarded as the interfering noise from the raw ECoG recordings. The values of these two statistical analysis methods have been demonstrated in both averaging and single trials (e.g., Cao et al. [15, 16]). In terms of single-trial analysis, wavelet-based time-frequency analysis is also used to assist the quantitative analysis of *Z*-score transformed power across different frequency bands. Whilst these statistical methods are not new the contribution of this paper is to integrate these methods with careful computational procedures and present a systematic study of the ECoG recordings for their LEP characterizations, which might offer some insights for the neurophysiological or clinical practice. To our best knowledge, we are in the first position or for the first time, to employ the statistical ICA tools to pain-related ECoG recordings. We describe the computational modeling and analysis in detail and present some interpretations and discussions from our experimental results. On the one hand, we strive to relate the results to the reported neurophysiological observations in the literature; on the other hand, we also pinpoint several interesting findings and observations in our single-trial data analysis.

## 2. DATA

### 2.1. Recordings

**Figure 1:**
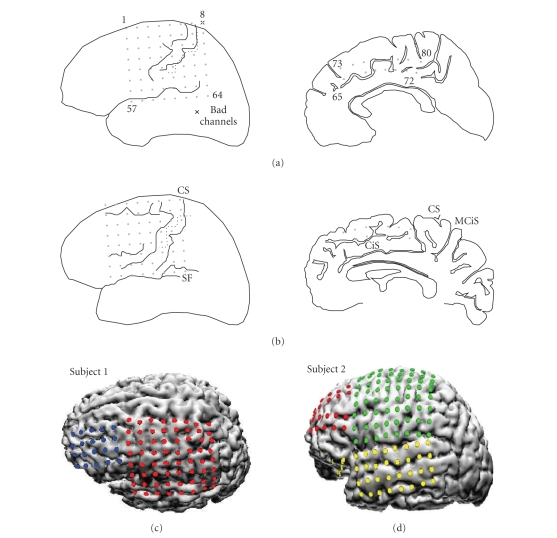
The implanted electrodes' layout; the somatosensory cortex that is associated with the sensation of the pain is located in the parietal lobe of the brain. (a) subject 1; (b) subject 2 (where CS and SF correspond to no. 8 and no. 64 channels, resp.); (c), (d) implanted grids imposed on the reconstructed 3D magnetic resonance images of two subjects. Note that the number of implanted grids shown on the 3D images is more than the number of the available channels shown in Table 1; because of the limitation in data acquisition, only a subset of the grids were selected (CS: central sulcus; SF: sylvian fissure; CiS: cingulate sulcus; MCiS: marginal CiS).

To obtain the ECoG recordings, special grid electrodes were implanted on the cortical surface of the subjects (i.e., patients for surgical treatment of epilepsy). The grid consisted of platinum-iridium circular electrodes (2.3mm diameter) with a center-to-center distance between electrodes of 1cm (Ad-Tech, Racine, Wis, USA). The LEPs were recorded with the implanted grid electrodes over the SI, PS, and MF regions; see Figure 1 for an illustration. During recordings, the subjects wore goggles and reclined on a bed, quietly wakeful with eyes open. Painful heat stimulation was delivered to the contralateral hand dorsum (contralateral to the grid) by a Thulium YAG laser (Neurotest, Wavelight Inc., Starnberg, Germany). The duration of each pulse was 1 millisecond and the beam diameter was 6mm. Laser energy level was determined to produce a painful sensation of 3-4/10 on a decimal scale (with 0 denoting no pain, and 10 denoting the most intense pain). The ECoG signals were recorded with sampling frequency 1000Hz. The recordings were carried out at the Johns Hopkins Hospital between 1999 and 2003 (Ohara et al. [7–9]). The protocol was reviewed and approved annually by the Institutional Review Board of the Johns Hopkins Hospital and all subjects signed an informed consent for the studies.

### 2.2. Subjects

For the purpose of presentation clarification and due to space limit, we have chosen two human subjects in the current study. The statistics of the recording setup regarding the selected two subjects are listed in Table 1. Specifically, the first subject was a 21-year old woman with medically intractable seizures since age 10; her neurological examinations and brain magnetic resonance images (MRIs) were normal. Subdural electrode grids were planted over the frontal-central-parasylvian cortex (no. 1–64 channels) and the medial wall of the left hemisphere (no. 65–80 channels). The second subject was a 21-year old man with complex partial seizures since age 4, whose MRI showed a small cavernoma in the right parietal lobe (contralateral to the side of the implantation). The ECoG signals were recorded from the left fronto-parietal lobe (64 channels) and medial frontal lobe (16 channels). All the signals were recorded with reference to one intracranial electrode.

### 2.3. Experimental paradigm

There are two types of experimental protocols designed for subjects: *attention/distraction* , and *intensity* . In the attention condition, the subject was asked to count the number of painful stimuli and to report both that number and the average pain intensity after each run of laser pulses; in the distraction condition, the subject read a magazine article and answered questions about it after the run. In these two conditions, constant level of laser intensity was used for the subject, and 38 laser pulses were delivered with an interstimulus interval that was randomly varied between 50 and 10 seconds within each run. Additionally, in the intensity experiment, varying levels of laser stimuli were delivered to the subject, and the subject was asked to rate the subjective pain sensation according to the decimal scale.

### 2.4. Filtering

Upon loading the raw ECoG recordings to the computer, the data were amplified and band-pass filtered at 0.1–300Hz (Astro-Med, Inc., West Warwick, RI, USA). Subsequently, we conducted a simple notch filtering procedure to filter out the AC components of power supply (60Hz).

## 3. MATHEMATICAL MODELING AND ANALYSIS

### 3.1. Generative model

The experimental data are assumed to be generated by a probabilistic generative model that is described by two equations as follows:

(1) $$x_{t}=\mu +Bz_{t}+\varepsilon_{t},$$

(2) $$z_{t}=As_{t},$$

where *t* denotes the time index. Equation (1) is essentially a factor analysis (FA) model, where ***z*** *_t_* ∈ *ℝ* ^*n*^ is the hidden variable called “factor,” the *m* × *n* matrix **B** is called the “loading matrix, ” **x** *_t_* ∈ *ℝ* ^*m*^ denotes the observed multi-channel signals measured in the electrodes, ***μ*** ∈ *ℝ* *^m^* denotes the constant mean vector that is often assumed to be zero, and **ε** *_t_* ∈ *ℝ* *^m^* denotes the additive uncorrelated noise that corrupts the measurements. Equation (2) describes a linear mixture model that is related to the blind source separation (BSS) problem of our interest, where ***s*** *_t_* ∈ *ℝ* *^N^* denotes the independent source signals originated from the brain, **A** denotes a linear mixing matrix that roughly models the mixing process and the stationary propagation or scattering effect within a short timescale (say, 200 to 600 mesc); and the mixed signals consist of the hidden factor ***z*** *_t_* obtained in (1). In the current setting of this paper, we assume *m* > *n* = *N*.

No doubt that the generative model described by (1) and (2) is somewhat oversimplified for the ECoG data. However, we believe that the instantaneous linear mixing model is rather reasonable at a short timescale and therefore can be used in the first step. In addition, we assume that matrices ***A*** and ***B*** are constant within the a short duration of measurements. Now, the statistical estimation problem is to infer the independent sources ***s*** *_t_* given the observed ***x*** *_t_*. We will tackle this problem via these two statistical tools as described below. Notably, similar methodology has been applied to MEG or EEG recordings with successes in some other real-life recordings (e.g., Cao et al. [15, 16]).

### 3.2. Factor analysis

Without loss of generality, we assume that *μ* = **0**, and the factor variables satisfy 𝔼[***z*** *_t_*] = **0** and 𝔼[***z*** *_t_* ***z*** *_t_* *^T^*] = **C**_**z**_, where **C**_**z**_ is the covariance matrix; and the noise is Gaussian distributed with zero mean and covariance matrix **Σ**, which we denote by **ε** ∼ *𝒩*(**0**,**Σ**). In light of (1), we have

(3) $$\begin{array}{c} 𝔼[x_{t}]=0,\\ 𝔼[x_{t}x_{t}^{T}]\equiv C_{x}=BC_{z}B^{T}+\Sigma . \end{array}$$

If **z** *_t_* is Gaussian distributed, then **x** *_t_* is also Gaussian distributed. If we further restrict that the factor **z** *_t_* is whitened, then **C_z_** = **I** (where **I** denotes the identity matrix); this assumption is reasonable since we can always scale the loading factors **B** to satisfy the original model equation. Typically, dim⁡(**x**) > dim⁡(**z**), therefore FA is also a dimensionality-reduction method. A close examination of our experimental multielectrode recordings indicates that there are strong correlations between adjacent electrodes, which therefore justifies the necessity of dimensionality reduction.

From a probabilistic point of view, we can write *p*(**z** *_t_*) = *𝒩*(**0**,**I**), then *p*(**x** *_t_*) = *𝒩*(**0**, **BB** *^T^* + **Σ**). Under the Gaussian assumption of the factor analyzer, the posterior probability *p*(**z** *_t_* | **x** *_t_*) is also Gaussian, with mean and covariance, respectively, defined by

(4) $$E\left[z_{t}|x_{t}\right]={\left(B^{T}\Sigma^{-1}B+I\right)}^{-1}B^{T}\Sigma^{-1}x_{t},$$

(5) $$\text{Cov}\left[z_{t}|x_{t}\right]={\left(B^{T}\Sigma^{-1}B+I\right)}^{-1}.$$

Now the goal of FA is to estimate the unknown matrices **B** and Σ, given the observed data {**x** *_t_*}. In the literature, two types of estimation procedures can be employed.

**Maximum likelihood estimation**

By deriving the log likelihood function (see the appendix) with respect to the unknown variables, we can use iterative optimization procedures, such as the gradient ascent or expectation-maximization (EM) algorithm, to obtain the optimal solution. Upon obtaining the maximum likelihood estimates of **B** and Σ, we can further calculate the hidden factor **z** *_t_* by (4).

**Least-squared estimation**

Given observed samples {**x** *_t_*}_*t* = 1_^*T*^, we can calculate the sample covariance matrix (assuming zero mean) and conduct its eigenvalue decomposition (EVD) as follows:

(6) $${\hat{C}}_{x}=\frac{1}{T}\sum_{t=1}^{T}x_{t}x_{t}^{T}=U\Lambda U^{T},$$

where **U** is the *m* × *m* orthogonal matrix that consists of eigenvectors as its column vectors, **Λ** is a diagonal matrix that consists of the diagonal entries as eigenvalues. Note that when the noise is zero or the noise is negligible and has a diagonal covariance matrix, then FA reduces to PCA as a special case. Upon PCA, we can empirically estimate the noise covariance. Let **U**_*n*_ denote an *m* × *n* matrix that consists of the first *n* dominant eigenvectors, then we can estimate the noise covariance by

(7) $$\hat{\Sigma }={\hat{C}}_{x}-U_{n}\Lambda_{n}U_{n}^{T},$$

and estimate the loading matrix by

(8) $$\hat{B}=U_{n}\Lambda_{n}^{1/2}.$$

Finally, the factor variable **z** *_t_* is produced by a linear transformation:

(9) $$z_{t}=Qx_{t},$$

where $Q={\left({\hat{B}}^{T}{\hat{\Sigma }}^{-1}\hat{B}\right)}^{-1}{\hat{B}}^{T}{\hat{\Sigma }}^{-1}$ . Note that in this case, the dimensionality of **z** *_t_* can be determined by PCA with dimensionality reduction, whereas the remaining components are considered to be “significant” in terms of variance or energy contribution.

### 3.3. Independent component analysis

Upon performing the model reduction using FA, we further aim to apply the blind source separation (BSS) approach, using the tool of ICA (e.g., Cichocki and Amari [27]), to recover the hidden sources in (2). Roughly speaking, ICA is built upon the assumption that the hidden sources in **s** *_t_* are mutually independent and subject to an instantaneous linear mixing.

There are many ICA/BSS algorithms available in the literature. To our interest, two kinds of batch (i.e., noniterative) ICA/BSS algorithms are considered.

**Time-domain method**

Specifically, we focus on the BSS algorithms based on generalized EVD of the time-delayed cross-correlation matrices or cumulant statistics, such as the SOBI (second-order blind identification) and JADE (joint approximate diagonalization of eigen-matrices) algorithms. These methods are fast and noniterative (thereby independent of the initial conditions). In our experiments, we have tried and compared the SOBI and JADE algorithms, and found that their results were qualitatively similar. However, JADE is more desirable and preferred since it incorporates higher-order statistics.

**Time-frequency method**

Specifically, the source separation criterion of this method is conducted in time-frequency domain based on joint diagonalization of the spatial time-frequency distribution (TFD). A representative example is the algorithm described by Févotte and Doncarli [28]. This method is more intuitively appealing (by taking into account of the information in both time and frequency) and has been demonstrated to be robust to noise.^3^

Notably, although the hidden factor **z** is whitened (with zero mean and unit variance), it is still likely that the mixing matrix is ill-conditioned, which thereby makes the estimation of its inverse (or Moore-Penrose pseudoinverse), the demixing matrix **W** = **A** ^−1^ (or **W** = **A** ^†^ ≡ (**A** ^*T*^ **A**)^−1^ **A** ^*T*^), rather difficult, especially in single-trial experiments. One way to overcome this problem is to conduct a two-stage ICA procedure. The essence of the two-stage ICA is as follows: the role of the first-stage ICA is “rough tuning,” which produces a guess (or poor estimate) of the ill-conditioned mixing matrix; and the final “fine tuning” job is accomplished by the second-stage ICA routine. The trick of such a two-stage ICA often helps to recover the hidden components in many ill-conditioned scenarios if it is not the case, the second-stage ICA simply produce improved or similar results as in the first-stage ICA.

The significance of the (uncorrelated or independent) components is determined by their relative energy (or variance). Physiologically, we believe those sources that have relative great energy are more meaningful in terms of repeatability. In practice, selecting the number of principal components is done by EVD followed by a threshold selection. In our experiments, five to eight principal components were typically selected, which account for about 97–99% of the total energy. Specifically, let **9** = diag{*λ*_1_,*λ*_2_,…,*λ*_n_} denote the diagonal matrix that contains the nondecreasing eigenvalues *λ*_1_ ≥ *λ*_2_ ≥⋯≥*λ*_n_≥ 0 , the number of significant components, *k*, is chosen according to the following criterion:

(10) $$k=\arg\;\underset{i}{\min}L_{i}\;\;\text{s}\text{.t}\text{.}L_{i}=\frac{\sum_{j=1}^{i}\lambda_{j}}{\sum_{j=1}^{n}\lambda_{j}}>Th,$$

in which the threshold Th was empirically set as 0.97; the nonnegative eigenvalue indicates the relative significance of specific component in terms of its energy contribution.

### 3.4. Identification of interested source by deflation

Let **y** ^(1)^ = **W** ^(1)^ **z** and **y** = **W** ^(2)^ **y** ^(1)^ denote, respectively, the first- and second-stage ICA unmixing equations, where **W** ^(1)^ and **W** ^(2)^ denote the associated unmixing matrices; then the final unmixed signals, **y** *_t_*, can be estimated as

(11) $$y_{t}={Wz}_{t}=W^{\left(2\right)}W^{\left(1\right)}z_{t},$$

where **W** = **W** ^(2)^ **W** ^(1)^ denote the global (combined) unmixing matrix.^4^ Notably, each column of **W** ^−1^ contains the relative strengths of a source component at the individual scalp electrodes, which can be used to identify the interested source component.

Given the estimated **y** *_t_* = [*y*_1_(*t*),*y*_2_(*t*),…,*y*_n_(*t*)]^*T*^, we can also reconstruct the *partial* hidden factor by projecting the *i*th component of **y** *_t_*, denoted by *y* *_i_*(*t*), backward onto the subspace^5^

(12) $${\hat{z}}_{t}=W^{†}{\left[0,\ldots ,0,y_{i}(t)\text{, 0, }\ldots ,0\right]}^{T}\equiv {\left[W^{†}\right]}_{i}y_{i}(t),$$

where [**W** ^†^]*_i_* denotes the *i*th column vector of the matrix **W** ^†^. Furthermore, we can reconstruct the specific source of interest in the observed data space (i.e., the scalp signals contributed merely to the *i*th source)

(13) $${\hat{x}}_{t}=Q^{†}{\hat{z}}_{t}=Q^{†}W^{†}{\left[0,\ldots ,0,y_{i}(t)\text{, 0, }\ldots ,0\right]}^{T}.$$

By projecting ${\hat{x}}_{t}$ to the original channels' positions (i.e., the 8 × 8 electrode layout), we essentially *identify* the source(s) of interest. It should be noted that the “source identification” here is only limited to the two-dimensional scalp surface, and does not refer to localization of the three-dimensional spatial position of the “voxel.”

In addition, in order to evaluate the *relative* contribution of every electrode to the extracted independent component (especially for the LEP), we need to consider the *joint* effect of ${\hat{x}}_{t}$ and **W**. For this purpose, we may also calculate the *weighted* estimate of the sensor space ${\hat{x}}_{t}$ as follows:

(14) $${\tilde{x}}_{t}=w_{i}^{T}⊙{\hat{x}}_{t}=[w_{i1}{\hat{x}}_{1}(t),w_{i2}{\hat{x}}_{2}(t),\ldots ,w_{in}{\hat{x}}_{n}(t{)]}^{T},$$

where ⊙ denotes the Hadamard (elementwise) product, *w* *_i_* = [*w* *_i_*_1_,*w* *_i_*_2_,…,*w* *_i_*_n_] denotes the *i*th row vector of the matrix **W**, and ${\hat{x}}_{t}={\left[{\hat{x}}_{1}\left(t\right),{\hat{x}}_{2}\left(t\right),\ldots ,{\hat{x}}_{n}\left(t\right)\right]}^{T}$ is the back-projected sensor space from the *i*th independent source via (13). As a distinction, we call the reconstructed ${\hat{x}}_{t}$ in the sensory space as “unweighted map” and the reconstructed ${\tilde{x}}_{t}$ in the sensory space as “weighted map.” Notably, because of the degeneracy of **W**, the “weighted map” is subject to the scaling and algebraic sign uncertainties.

### 3.5. Time-frequency analysis

In addition to analyzing temporal signals, we also resort on time-frequency analysis tools (such as the short-time Fourier transform, or Wigner-Ville distribution, and wavelet transform) to extract more information for quantitative comparisons. Specifically, wavelet analysis is appealing and considered superior to the short-time Fourier transform for nonstationary signals, including EEG (e.g., Mallat et al. [29]; Tallon-Baudry et al. [30], Düzel et al. [31], Mouraux et al. [12], Ohara et al. [7]). Here, we choose the continuous wavelet transform for our purpose because of its adaptive time-frequency analysis via multiscale decomposition. However, because of the *uncertainty principle* , in order to obtain a good frequency resolution, sufficient time samples are required. In the experiments, we will use the Wigner-Ville distribution for an illustration purpose, while in the quantitative analysis we will use the continuous wavelet transform.

For a temporal signal *x*(*t*) (i.e., the raw recordings from one electrode channel), the power of its continuous-time wavelet transform is described by

(15) $$X(t,\omega_{0})=|x(t)*\psi (t,\omega_{0}{)|}^{2},$$

where ∗ denotes convolution product between the signal and the mother wavelet function, and *ψ*(*t*,*ω*_0_) is a complex-valued Morlet mother function:

(16) $$\psi (t,\omega_{0})=(\sigma^{2}\pi {)}^{-1/4}\exp(\frac{-t^{2}}{2\sigma^{2}})\exp(j2\pi \omega_{0}t),$$

where $j=\sqrt{-1}$, and *σ* is the bandwidth parameter. The width of the Morlet wavelet, defined by 2*π* *σ* *ω*_0_, is set to 7 in our study.^6^ The central frequency *ω*_0_ ranges from 1 to 60 Hz in steps of 1 Hz. To analyze the specific temporal window of interest, we select a 100-millisecond prestimulus period and a 500-millisecond poststimulus period, with a total window length 600 milliseconds.

**Figure 2:**
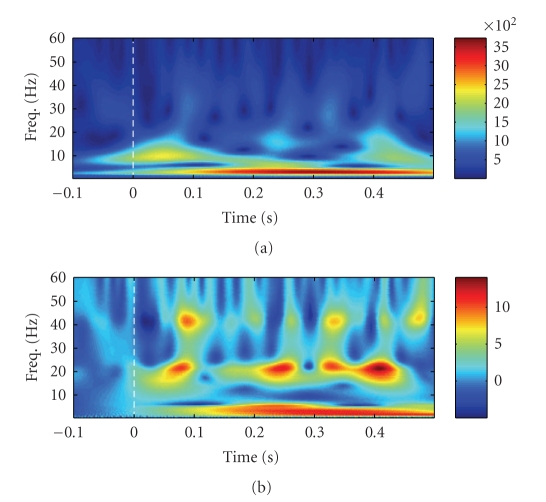
The original (upper panel, unit *μ* **V** ^2^) versus *Z*-score transformed (bottom panel, unitless) wavelet scalogram of one selected channel in a single trial (subject 2, attention task, laser intensity 720mJ). The white dash lines indicate the laser stimulus onset. As seen, the ERS and ERD are highlighted more clearly by the *Z*-score transformation given by (18).

To compare the power change between the prestimulus and poststimulus periods, we need to introduce some “relative” measures to obtain a baseline for the poststimulus power. This is important because we are not interested in the “absolute” power statistic *per se* , but interested in the stimulus-induced relative power change. In the literature, the measure of event-related band power change (ERBP) was defined as (e.g., Ohara et al. [7])

(17) $$ERBP(t,\omega_{0})=10\log(\frac{X(t,\omega_{0})}{m(\omega_{0})})(dB),$$

where *m*(*ω*_0_) denotes the *median* power envelope during the prestimulus period. Alternatively, we can use another measure, which we refer to as “ *Z*-score transformed poststimulus power,” by using the *Z*-score transformation (e.g., Browne and Cutmore [20]):

(18) $$\tilde{X}(t,\omega_{0})=\frac{X(t,\omega_{0})-\mu (\omega_{0})}{\sigma (\omega_{0})},$$

where *μ*(*ω*_0_) and *σ*(*ω*_0_) are, respectively, the *mean* and *standard deviation* of the power in a specific channel band (with center frequency *ω*_0_) during the prestimulus period. The motivation of (18) is to introduce baseline power values across different frequency bands for the poststimulus power statistics, which are used for standardized comparisons. In doing so, the low-amplitude component at high frequency will be highlighted, which also makes the time-frequency atom in the *gamma* (>32 Hz) band more visible. See Figure 2 for an illustrative example. Note that the *Z*-score power value can be negative; the positive values indicate the *event-related synchronization* (ERS), and the negative values indicate the *event-related desynchronization* (ERD), both between the prestimulus and poststimulus periods. Hence, the *Z*-score transformation provides a clearer understanding of the time-frequency map (in terms of relative power change).

In some cases, the resulted *Z*-score transformed poststimulus power will be converted to a two-dimensional time-frequency distribution map, denoted by *E*(*t*, *ω*), and further normalized to unity such that *∬* *E*(*t*,*ω*)*d* *ω* *d* *t* = 1, which we refer to as the normalized power. In doing so, each time-frequency atom can be interpreted by a nonnegative probability in the time-frequency plane.

## 4. COMPARATIVE EXPERIMENTS FOR AVERAGING TRIALS

We first apply the above described computational procedure and statistical tools for averaging trials, the signal-trial experiments will be treated later in more detail. The experimental results reported in this section will be illustrated for subject 1; two kinds of conditions, counting and reading, are considered.

### 4.1. Extraction of laser-evoked potentials

**Figure 3:**
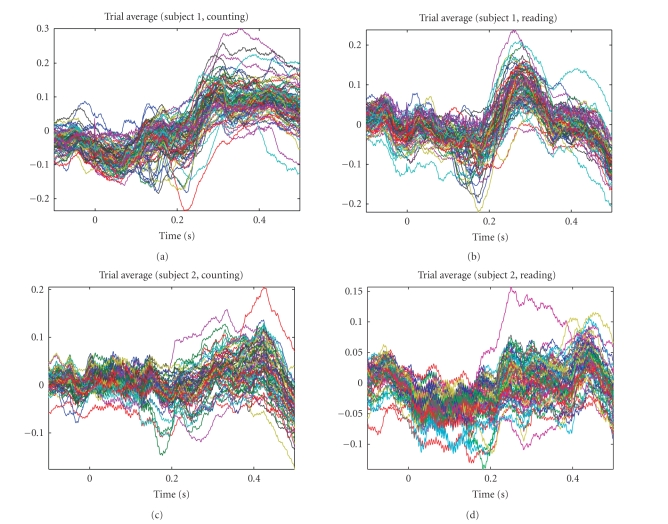
The averaging waveforms (arbitrary scaling) from averaging trials for both subjects in two tasks. As seen, the averaging evoked potentials are not clearly evident in these plots.

First, we aim at extracting LEPs for the averaging-trial experiments. Specifically, according to the laser onset tag, the ECoG recordings (of all channels) were averaged upon the total number of trials at each run. By doing so, the effect of the visual or muscle artifacts may be greatly reduced. However, it is difficult to identify the LEPs from the averaging ECoG waveforms of all channels (see Figure 3). Not only the peaks of the LEPs are less evident, the averaging waveforms still suffer from noise and artifact corruption.

**Figure 4:**
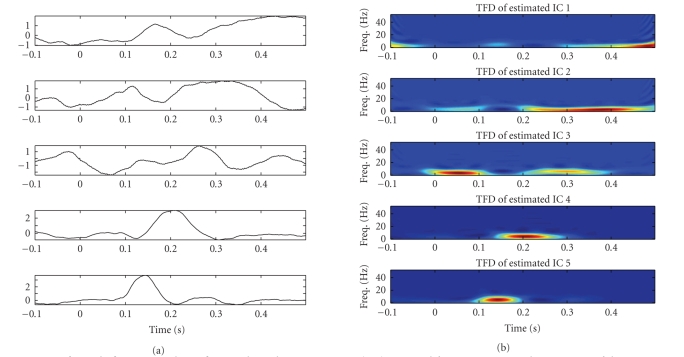
*Left panels:* five estimated significant independent components (ICs) extracted from averaging-trial experiment of the counting task (attention situation) for subject 1. *Right panels:* the associated time-frequency distribution (TFD) maps.

**Figure 5:**
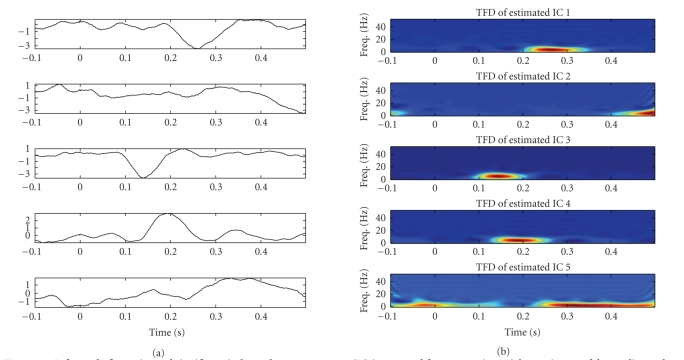
*Left panels:* five estimated significant independent components (ICs) extracted from averaging-trial experiment of the reading task (distraction situation) for subject 1. *Right panels:* the associated time-frequency distribution (TFD) maps.

To overcome these issues, we then apply the statistical tools (FA and ICA) to further process these trial-averaging signals. In the averaging-trial experiments for subject 1, we selected five independent components for the purpose of extracting LEPs. These five independent components are considered to be “significant” because they contribute mostly to the averaged ECoG data in terms of variance or energy.^7^ Due to the averaging/smoothing effect, one-stage ICA procedure (with the JADE algorithm) was found typically sufficient in the experiments^8^ The experimental results for the subject 1, in the time domain as well as in the time-frequency domain, are illustrated in Figures 4 and 5. As observed in the figures, we can extract typical peaks around 150 milliseconds and 200 milliseconds, which might correspond to the hypothetic N2 and P2 peaks (or N2^∗^ and P2^∗^^∗^) of LEPs, which we also refer to as N150 and P200, (a), 5(a), 13, 15(a), and 21. Please check. respectively; the other components can be viewed as other significant independent spontaneous brain activities. These findings were confirmed in both attention (counting) and distraction (reading) conditions.

**Figure 6:**
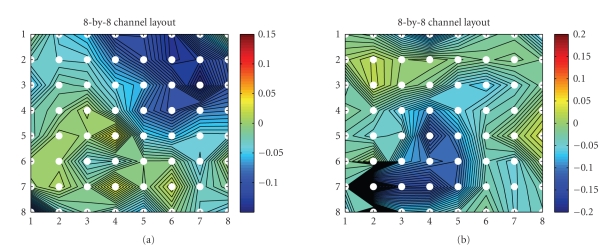
Source identification in the averaging trial of subject 1: the back-projected 8 × 8 (first 64 channels) scaled amplitude contour map of the LEP peak at N150 (the 5th independent source at 150 milliseconds, left panel) and P200 (the 4th independent source at 200 milliseconds, right panel) in the counting task (attention condition).

**Figure 7:**
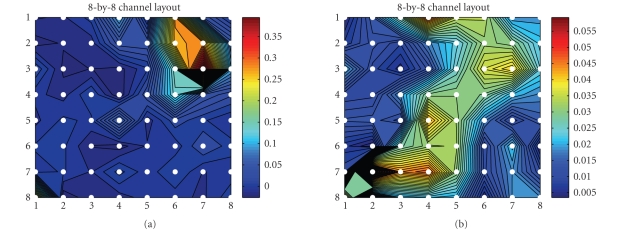
*Left panel:* the “weighted” map of LEP-N150 (compared to the “unweighted” map the left panel of Figure 5) from (14). *Right panel*: the back-projected 8-by-8 (first 64 channels) power (i.e., the absolute value of the amplitude) contour map of the two LEPs, N150 and P200, averaged between 120 milliseconds to 240 milliseconds (subject 1, attention condition).

**Figure 8:**
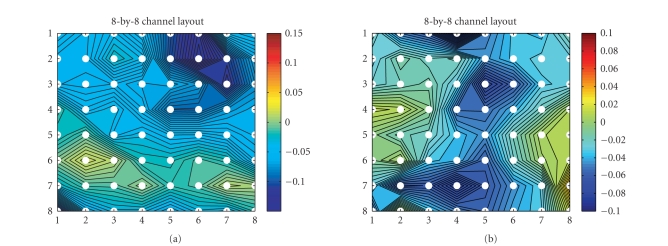
Source identification in the averaging trial of subject 1: the back-projected 8-by-8 (first 64 channels) scaled amplitude contour map of the LEP peak at N150 (the 3rd independent source at 150 milliseconds, left panel) and P200 (the 4th independent source at 200 milliseconds, right panel) in the reading task (distraction condition).

**Figure 9:**
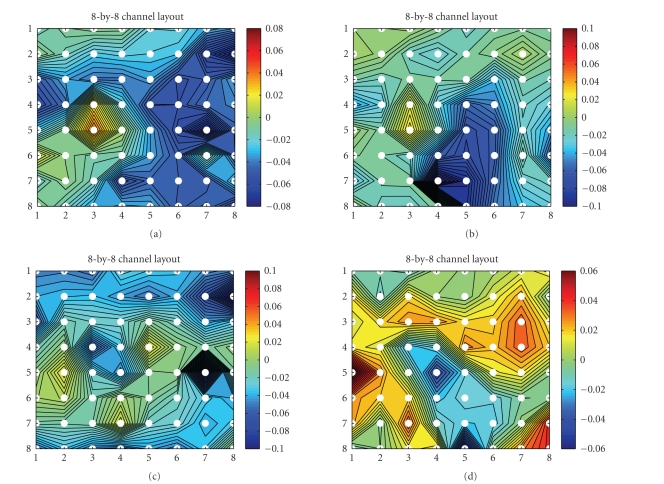
Source identification in the averaging trial of subject 2: the back-projected 8-by-8 (first 64 channels) scaled amplitude contour map of the LEP peak at N2 and P2 in the counting (top 2 panels) and reading (bottom 2 panels) tasks.

Next, we conduct the task of LEP source identification. This is done by back-projecting the *i*th independent component (i.e., the estimated LEP) back to the observed sensor space. Specifically, the power contour maps of N2 (N150) and P2 (P200) under the attention and distraction conditions are illustrated in Figures 6 and 8, respectively. The results are qualitatively close (but not identical) to the previous study (Ohara et al. [7, 8]), in which the LEP peak was found over the interhemispheric (medial) surface. We also plot the *combined* contributions of the power contour map for N150 and P200, averaged from 120 milliseconds to 240 milliseconds, as shown in Figure 7. As seen in the figure, in the LEP-N2 (i.e., N150), the greatest brain activities happen around the vertex (Cz)—the upper right corner of the 8 × 8 electrode layout (see Figures 1, 6, and 7), these observations were consistent with our early result (Ohara et al. [7, 8]), as well as other independent findings using EEG and fMRI with a similar setup (e.g., see Figure 1 of Iannetti et al. [5]). Similarly, we also obtained the LEPs' mappings for subject 2 (see Figure 9).

### 4.2. Relative power

**Figure 10:**
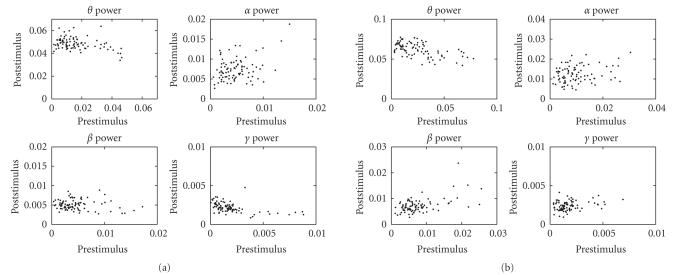
The scatter plots of prestimulus and poststimulus power comparisons in averaging trials for 89 channels (subject 1, left: counting task, right: reading task).

**Figure 11:**
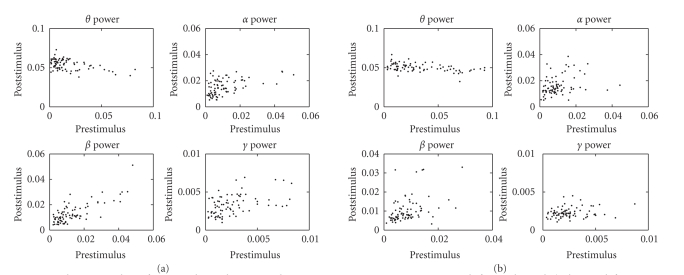
The scatter plots of prestimulus and poststimulus power comparisons in averaging trials for 80 channels (subject 2, left: counting task, right: reading task).

We compare the time-frequency distribution (TFD) power between the prestimulus and poststimulus periods. The averaged total power (per channel) and the averaged power (per channel) of specific frequency bands, including *theta* (4–7.5 Hz), *alpha* (8–12Hz), *beta* (12.5–32Hz), and *gamma* (32–60Hz), are all calculated. In Table 2, we summarize the statistics of two subjects under the attention (counting) and distraction (reading) conditions. The corresponding scatter plots of prestimulus and poststimulus power (of selected frequency bands) of all channels are shown in Figures 10 and 11.

From Table 2, several observations are noteworthy.

- The power in the poststimulus period is generally greater than that in the prestimulus period, which is obviously evidenced in terms of total power, *θ* and α power.
- The *θ* power increase (or ERS) is relatively more pronounced in the attention condition than in the distraction condition.
- The *β* power remains roughly the same level after the laser stimulus, regardless of the undertaken tasks.
- The *γ* power is typically small in all conditions, with slightly greater value in the attention condition than in the distraction condition.

It is noteworthy that the above observations are consistent with the findings reported in neuroscience and neurophysiology (to name a few, Bromm and Lorenz [1], García-Larrea et al. [2], Ohara et al. [7]). Although the statistics summarized in Table 2 are calculated based on the averaging trials, statistical test (see the next subsection) on single trials also reveals statistical significance.

### 4.3. Statistical hypothesis testing

In order to evaluate the results of the averaging trials, we conduct some statistical hypothesis tests in order to confirm the “statistical meaning” of the extracted LEPs. This procedure is necessary because the result of the extracted LEPs in the averaging trials does not tell anything *in statistical sense* about each single trial; namely, we need to be sure if the results we are tempted to interpret are due to random effects from averaging, or due to the consistent causality in all or most of individual single trials.

Two popular hypothesis testing methods we consider here are the ANOVA (analysis of variance, or *F*-test) and Mann-Whitney test (or *U*-test). In our experiments, we first use the Mann-Whiteny test to calculate the so-called *P*-value. Second, we also apply a logarithm transformation of the raw samples in attempt to obtain the Gaussianity (i.e., the raw samples are lognormal distributed, as confirmed by the Shapiro-Wilk test), and then apply the ANOVA to calculate the *P*-values.^9^

**Figure 12:**
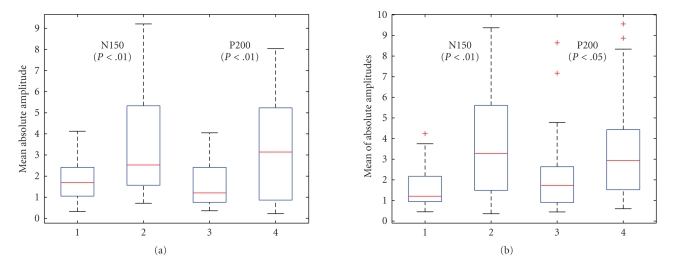
Boxplots of the absolute value of raw samples, together with their Mann-Whitney test *P*-values on the counting (left panel) and reading (right panel) tasks (subject 1, laser intensity 720 mJ).

To conduct the statistical tests, we apply the estimated unmixing matrix **W** from the averaged trial to each single trial; then we obtain the *surrogate* “single-trial LEP”^10^ for individual single trials, for either LEP-N150 or LEP-P200. For a specific LEP component, we expect that there is a consistent and significant difference between the prestimulus and poststimulus periods in terms of their absolute values. In our case, the statistical test was conducted in the time-frequency domain. For instance, for the LEP-N150 (or LEP-P200), according to its time-frequency map, we empirically choose a window around the maximum power value (i.e., the magnitude) in the time-frequency map,^11^ and further conducted the Mann-Whitney test for each extracted LEP component in all single trials, in which the comparison was done in the time-frequency domain. Specifically, we compared the *average mean* of the power value inside the time-frequency window centered around the maximum point (which in the time domain corresponds to the extracted LEP peak) with that of the prestimulus period (with the same time-frequency window size), both averaged across all frequency bins. Consequently, we may expect that the signal amplitude in the region of interest is significantly greater than that of the baseline. The statistical hypothesis testing results are summarized in Table 3, and the corresponding boxplots are shown in Figure 12. As seen in the table, the *P*-values of *U*-test are all smaller than .05, and three of them are much smaller than .01, consequentially, they are statistically significant. For the sake of completeness and sanity check, we also calculated the *P*-values that are not associated with the LEPs, (i.e., the other independent components extracted from the averaged-trials), we have consistently observed that their *P*-values are greater than .2 (around .2*∼*.6); hence, we can conclude that these non-LEP components obtained in the averaged trials are ascribed by the random effect that is not consistent in each single trial.

## 5. QUALITATIVE AND QUANTITATIVE ANALYSES OF SINGLE-TRIAL RECORDINGS

The averaging-trial experiments and statistical tests described above present an informative baseline and guideline for further single-trial experiments. As we mentioned earlier, it is well known that by averaging the ECoG recordings, we might lose some valuable information due to cancelation. For this reason, single-trial experimental findings would be also interesting. Nevertheless, single-trial analysis is more challenging because of the random background activities and artifacts; hence, obtaining consistent yet interpretable results is quite difficult. To succeed, we may require additional care or more sophisticated processing. Table 4 lists the operation comparisons between the averaging and single-trial analyses at each stage of procedure.

Notably, in contrast to the averaging-trial experiments in which the artifact effects are greatly reduced, strong artifacts may exist in the single-trial experiments. In practice, artifacts (often with low-frequency components) are sometimes observed by visual inspection. In this case, we will be cautioned about using these “bad” channels. A simple solution is to discard them or average with their neighboring channels. Selection of bad channels is often assisted with the reference of averaging trials. For instance, channels with extremely high amplitude and low frequency are generally regarded as eye movement artifacts. Since the FA/ICA statistical methods described above can somehow reduce these effects, hence only those channels with obvious artifacts were removed in the experimental procedure.

In the sequel, we will conduct qualitative and quantitative comparisons of single-trial recordings for different measurements listed in Table 1.

### 5.1. Setup

In single-trial experiments, the number of independent components usually varies from trial to trial (for the purpose of extracting LEPs), and we typically choose the number between 5 and 8. This is because in individual single trials, some small-amplitude but potentially important components at high frequency may play a crucial role, which is also interesting to observe. For the same purpose, we will use the two-stage ICA procedure (JADE algorithm followed by TFD joint diagonalization) described earlier in Section 3.

Upon extracting the LEP of interest, we further identify the LEP localization in the sensor space and focus on the analysis on *one* specific channel (in contrast to the analysis of *all* channels in the averaging trials). Specifically, we will examine the single-trial recordings under attention and distraction conditions, as well as the statistics of the LEP attributes (latency and amplitude) with varying pain levels (i.e., given different laser intensities).

### 5.2. Single trials versus averaging trials

In the single-trial experiments, we apply the above-described procedure with the goal of extracting the LEPs under different conditions, and the results obtained in the averaging trials are considered to be the baselines for qualitative comparison.

**Figure 13:**
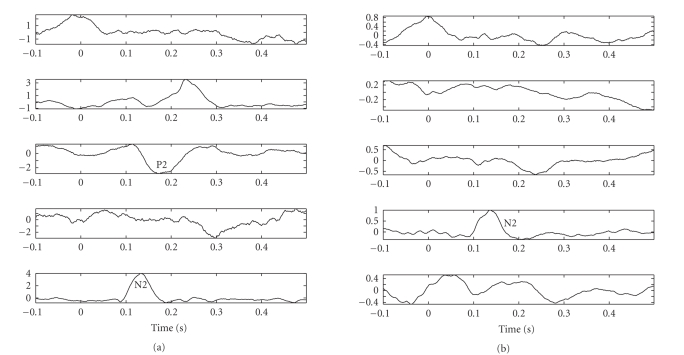
The extracted independent components (including LEPs) in signal-trials experiments for counting (left) and reading (right) tasks (subject 1, laser intensity 720 mJ).

Typically, not all single trials have good quality recordings compared with the averaging trials. Here we show a few successful examples that are capable of identifying the markers of the LEPs. Notably, in our experiments, it was observed that the LEP-N2 can be easily identified, while the LEP-P2 is more difficult to separate. See Figure 13 for two illustrations under different setting conditions.

**Figure 14:**
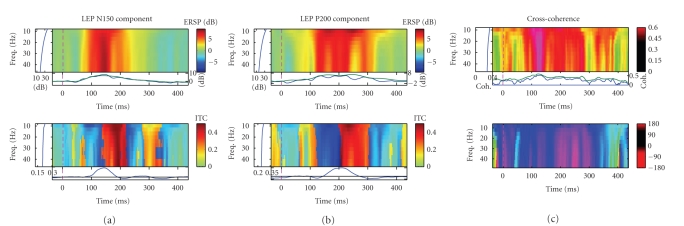
*Left and middle panels:* event-related (log) power spectral perturbation (ERSP, in dB, top row) and inter-trial coherence (ITC, from 0 to 1, bottom row) changes time locked to the LEP components in single trials (subject 1, attention task). *Right panel:* cross-coherence between LEP N150 and P200, with magnitude plot (from 0 to 1; top row) and phase plot (from −180 to 180 degree; bottom row).

In order to evaluate the variability between different single trials, we apply the estimated demixing matrix **W** obtained from averaging trials to all individual single trials, by which we obtain a set of LEP components for N150 and P200 (one pair for each single trial). Furthermore, we may use the available tools of the EEGLAB toolbox (http://www.sccn.ucsd.edu/eeglab; Delorme and Makeig [32], Delorme et al. [33], Makeig et al. [25]) to visualize the event-related spectral perturbation (ERSP) and the intertrial coherence (ITL) for the specific LEP components, as well as the cross-coherence between the independent LEP components. Specifically, the ERSP shows the spectral power change from prestimulus baseline (in dB) relative to the stimulus onset; and the ITL measures the consistency or reproducibility of the phase of stimulus-locked trial activity in the selected independent components. For instance, see Figure 14 for an illustrative example of two LEP components obtained from the attention task (recalling Figure 4). As seen in the figure, the cross-coherence magnitude (from 0 and 1) indicates the degree of synchronization between two independent LEP components, and the cross-coherence phase (from −180 to 180 degree) indicates that the LEP-N150 component is leading ahead of the LEP-P200 component.

### 5.3. Attention versus distraction

**Figure 15:**
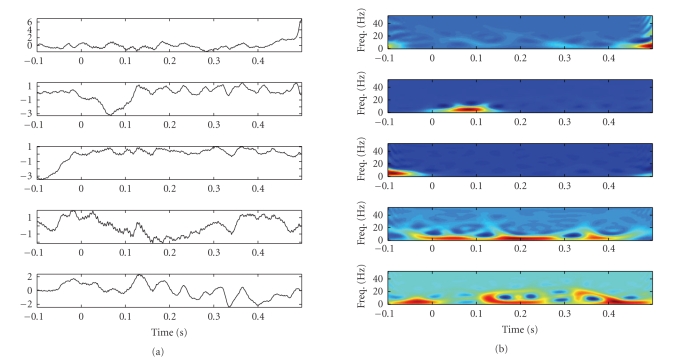
*Left panels:* the 5 estimated sources extracted from a single-trial experiment of the reading task (subject 2). The 5th independent source contains typical alpha waves. *Right panels:* the corresponding time-frequency representation.

For subjects 1 and 2, consistent alpha waves were found among many (but not all) single trials in the reading task (i.e., distraction condition); whereas in the counting task (i.e., attention condition), the significant alpha component was not observed in most of single trials. In some reading tasks, no obvious LEP was identified, while the dominant alpha waves can be observed. See Figure 15 for an illustration. In such cases, since there are no clear LEP peaks being observed, it remains an open question that whether this phenomenon is ascribed to “ *habituation to the pain* ” or “ *loss of attention* .” The reason that alpha rhythms appear frequently in the reading task might be due to the fact that the subject was in a relatively relaxed mood (especially compared with the counting task).

**Figure 16:**
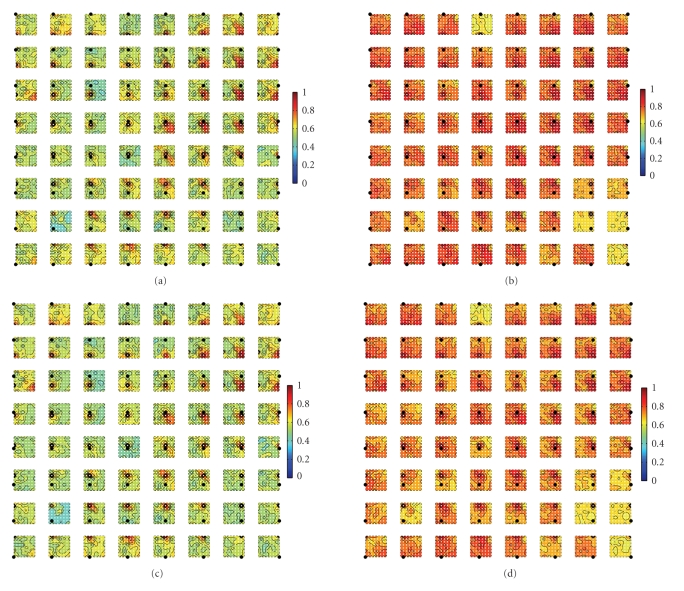
Pairwise coherence maps between the first 64 channels (subject 1, laser intensity 720 mJ) averaged over all single trials within a duration of 800 milliseconds in poststimulus period. (a) *alpha*-range coherence in the counting task; (b) *alpha*-range coherence in the reading task. (c) *beta*-range coherence in the counting task; (d) *beta*-range coherence in the reading task.

In addition, we also measure the coherence of signal-trial ECoG data under different conditions. In Figure 16, the coherency of alpha (8–12Hz) and beta (12.5–32Hz) bands between pairwise channels during the poststimulus period is illustrated. In order to visualize the coherency, putting all connections in one plot will be informative. Specifically, the complete 8-by-8 layout illustrates the first 64 electrodes' positions; at each electrode's position, we also plot aN 8-by-8 contour plot that represents the pairwise coherence between a specific electrode and the other electrodes, in which the specific electrode is marked by a relatively big filled circle. As seen, typically there is strong coherence in the range of neighboring electrodes. Comparing Figure 16(a) with Figure 16(b), and Figure 16(c) with Figure 16(d), we can observe that there is stronger coherence in the alpha and beta bands in the distraction condition than in the attention condition.

### 5.4. LEP-component power versus laser intensity

For the same human subject in a series of single trials, it is expected that varying the level of stimuli (by changing the laser intensity), the *amplitude* and *latency* of the LEPs will consequently vary, so does the *power* of the LEP components in the time-frequency map. For this purpose of analyzing the power of LEP components at difference frequency bands, we have conducted quantitative and comparative analysis for subject 2 under varying controlled conditions.

**Figure 17:**
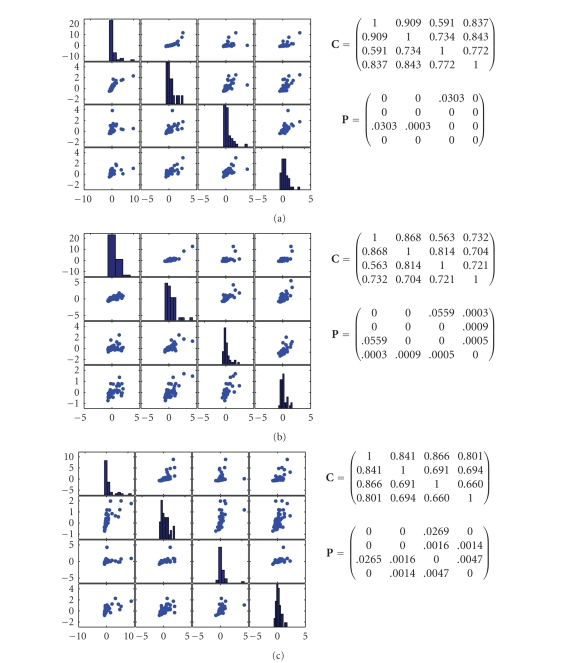
The scatter plots (using the MATLAB function “plotmatrix”) of the *Z*-score transformed power for the *theta, alpha, beta* , and *gamma* bands: (a) 480 mJ, (b) 640 mJ, (c) 800 mJ, each based upon 40, 44, and 40 single trials, respectively. At each panel, the diagonal plots show the histograms of *Z*-score power of the associated frequency bands (from left to right, theta, alpha, beta, and gamma); the off-diagonal plots show the scatter plots of *Z*-score power across different frequency bands. Matrix **C** contains the correlation coefficients, and matrix **P** contains the associated *P*-values from the student's *t*-test.

The power statistics are summarized in Table 5. It should be noted that the power values in Table 5 refer to the “ *Z*-score transformed” poststimulus power according to (18), and all the values are averaged over the total number of trials in each run. The statistics are calculated for the first 64 channels including the one that has the highest power contribution (no. 14 channel) at each trial. The scatter plots of the *Z*-score transformed power for *theta, alpha, beta*, and *gamma* bands are shown in Figure 17. In the off-diagonal subplots, the scatter plots of cross-band power are shown; whereas the diagonal subplots show the histograms of the power distribution in the relative frequency bands. In each subplot, the correlation coefficient between the power across different frequency bands is also calculated (stored in matrix **C**), as well as the associated *P*-values for the student's *t*-test (stored in matrix **P**). As seen, with different levels of laser intensities, the *Z*-score transformed power across different bands is correlated to certain degree: as the laser intensity increases, the degree of correlation at certain frequency bands (e.g., between *theta* and *alpha*) tends to decrease. A cut-off correlation coefficient of 0.7 was considered as a sign of significance. Each *P*-value indicates the probability of testing the hypothesis of no correlation, or the probability of getting a correlation as large as the observed value by random chance, when the true correlation is zero. If *P*(*i*, *j*) is small (say, less than 0.05), then the correlation **C**(*i*, *j*) is statistically significant.

From our data analysis, several observations are noteworthy.

**Figure 18:**
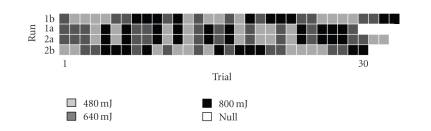
The graphical illustration of the laser intensity presentation orders at different runs (1a, 1b, 2a, and 2b). Note that the combined 62 trial sequences of “1a + 1b” and “2a + 2b” are of identical order.

**Figure 19:**
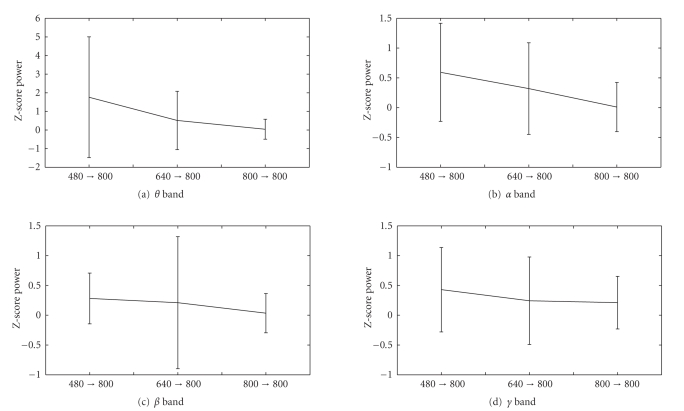
The *Z*-score power comparison of three different hysteresis effects. (a) *theta* band, (b) *alpha* band, (c) *beta* band, (d) *gamma* band.

- Compared to the prestimulus period, the power across different frequency bands in the poststimulus period mostly (or in majority) increases, as evidenced by the *positive* mean values of the *Z*-score transformed (relative) power, although their standard deviations are relative large.
- In one specific run, the general trend is that the *Z*-score transformed *θ* power increases as the laser intensity increases; it seems that no general rule can be found for α, *β*, and *γ* power among our experiments.
- In different runs (i.e., 1a, 1b, 2a, 2b), the mean power statistics with the same laser intensity often vary. This is not unreasonable because in each run the conditions of the subject may be different; in addition, the (random) order for presenting the laser stimuli is also different in each run (see Figure 18), their overall effects (say, e.g., between 480 → 640 → 800 and 640 → 800 → 480) would be certainly distinct. Such a “hysteresis” phenomenon is well known in psychology and psychophysics. In an effort to investigate this phenomenon, we take the 800mJ intensity level as an example. According to Figure 18, the total numbers of 480mJ, 640mJ, and 800mJ preceding 800mJ are 10, 16, and 14, respectively. In order to compare their effects on the *Z*-score power, we calculate the means and standard deviations of different frequency bands under these three different conditions (namely, 480 → 800 , 640 → 800 , 800 → 800 ), and the results are shown in Figure 19. It is interesting to observe from the figure that their *Z*-score power statistics are quite different especially at the low-frequency (theta and alpha) bands. Generally, the *Z*-score power are highest for 480 → 800 , followed by 640 → 800 , and then lowest for 800 → 800 this is not surprising considering the sensation habituation effect. Statistical tests show at the theta and alpha bands, the pairwise comparison of *Z*-scored power among three conditions is statistically significant (ANOVA, *P* < .01).

### 5.5. LEP amplitude and latency versus laser intensity

Consistent with the previous studies (Ohara et al. [7, 8]), the peak amplitudes were measured from the baseline value, which was defined as the averaged value during the prestimulus period. Latencies were measured as the time of the peak amplitude (except for the artifact) for each component; and peak was regarded as significant when the peak amplitude was above the mean ± SD prestimulus level. However, in the previous studies, peak amplitudes and latencies were both measured from reproducible, averaged waveforms; here, we attempt to measure the latencies from single trials, while the amplitude will still be measured from averaging (over the trials at each run) because of its strong randomness; and the standard deviation of the amplitude estimate is calculated based on 4 independent runs among the recordings. In the meantime, we will focus the measurements on the first 64 electrodes (channels) for the primary somatosensory (SI) region, while the analyses for the parasylvian and medial frontal (MF) regions are ignored here. As observed in our experiments (Table 6), the averaged amplitudes of the LEPs (for both N2 and P2) increase as increasing laser intensity, except for one case of P2 under the 800mJ condition; however, the mean statistic is also accompanied with a relatively large standard deviation, which reflects the random variations of measurement and/or subject conditions.

**Figure 20:**
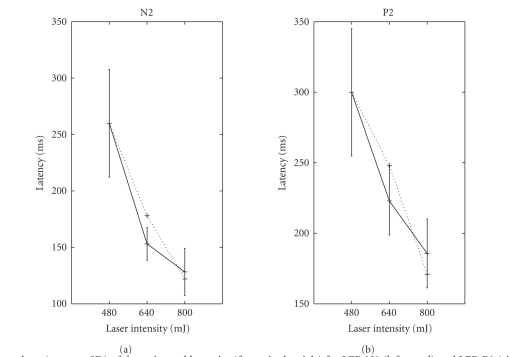
The error bars (mean ± SD) of the estimated latencies (from single trials) for LEP-N2 (left panel) and LEP-P2 (right panel) with varying laser intensities. The dotted lines indicate the estimated latencies from the averaging trials.

In our single-trial experiments, it was found that the latencies of the LEPs vary from trials to trials, evidenced by a large standard deviations (see Figure 20). In addition, by varying the laser intensity, the LEP-N2 and LEP-P2 also exhibit different attributes in terms of latency and amplitude. The corresponding statistics are summarized in Table 6 and Figure 20. Specifically, several observations are noteworthy.

**Figure 21:**
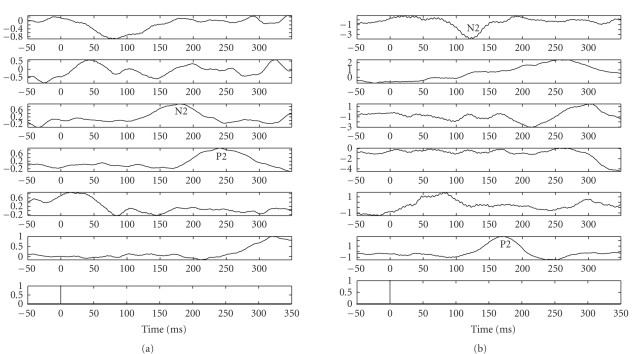
The 6 estimated sources extracted from averaged trials (subject 2) with different laser intensities (left panel: 640mJ, right panel: 800mJ).

- As seen in Table 6, the stronger is the laser intensity, the sooner the LEP appears; namely, the value of the LEP latency is smaller. See Figure 21 for two illustrative results.
- When the laser intensity is small (e.g., 480mJ), it is quite difficult to extract the LEP (either one or two) with the available ICA technique. This is partly because the LEP is so weak that it is overwhelmed in the background “noise” (brain activities). Indeed, it is even difficult to identify the peaks via visual inspection from the averaging recordings.

Generally, the amplitude of the LEP is a reflection of the sensation of the pain. Although it seems difficult to discover quantitative relationship between the intensity of the laser beam and the amplitude/latency of the LEPs, it is qualitatively clear that there exists correlation between them, especially when the intensity difference is large. This phenomenon might serve as a useful evaluation tool in the clinical practice.

To evaluate the statistical significance of the LEP peak amplitude and latency, we also conduct statistical tests based on their single-trial measurements. We first conduct a robust linear regression fit (using the MATLAB function “robustfit”) between the laser intensity value (regression variable) and the measure of interest (amplitude or latency of the LEP), and then obtain Pearson's correlation statistic *r*. Next, we calculate the *t* statistic as follows:

$$\begin{array}{l} t=\frac{r\sqrt{\ell -2}}{\sqrt{1-r^{2}}}, \end{array}$$

where ℓ denotes the number of regression sample pairs. From the *t*-statistic, we can further evaluate the statistical significance (i.e., *P*-value) from the *t*-table. In our case, we found the linear fit for LEP's latency is significant (*r* = 0.87, *P* < .05); however, the linear fit for LEP's amplitude is not significant. 

### 5.6. Subjective sensation versus laser intensity

**Figure 22:**
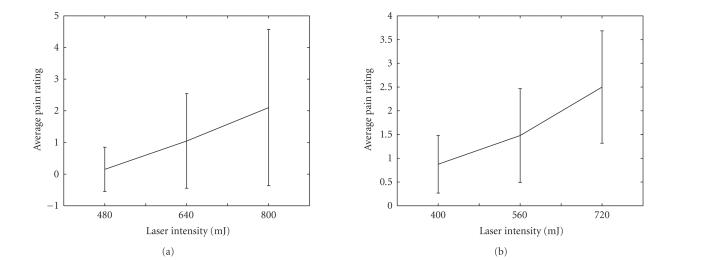
Pain sensation rating at different levels of laser intensity (left panel, averaged over all 124 single trials for subject 2). This is compared with another subject (right panel, averaged over 124 single trials, data from Ohara et al. [34]).

Finally, we follow the procedure of Ohara et al. [34] to analyze the relationship between the subjective sensation (in terms of pain rating) and the laser intensity. Specifically, the subject was asked to rate the pain level in decimal scale (0 no pain, 10 the most intense pain sensation). The mean and standard deviation statistics are calculated based on all single trials given three different laser intensities, as shown in Figure 22. Generally, it is seen that the average subjective pain sensation increases as the level of the laser intensity increases. Statistical tests show significant sensation differences between different levels of laser intensities (ANOVA, P < .001 between 480 mJ and 800 mJ; P < .05 between 640 mJ and 800 mJ). Moreover, we also evaluate the correlation between subjective sensation and LEP amplitude; however, no significant correlation was observed between the pain sensation rating and LEP amplitude for subject 2. We suspect this is partially due to the large variations among the subjective pain rating, even with the same laser intensity (specifically, the mean ± SD of the pain rating value for laser intensities 480 mJ, 640 mJ, and 800 mJ are 0.15 ± 0.70, 1.05 ± 1.49, and 2.10 ± 2.47, resp.). Although our data here seem to suggest that the subjective pain sensation and the objective LEP attribute observation might not be necessarily correlated, we should also be cautioned that the pain is a very complex sensation and is susceptible to many human factors and experimental conditions. Verification of any claim in this matter require more data and careful analysis.

## 6. DISCUSSION AND CONCLUSION

In this paper, we have used the statistical tools of FA/ICA for extracting and analyzing the LEPs. To our best knowledge, the statistical analysis and quantitative results reported here are among the new (if not the first) reports that apply sophisticated and systematic statistical analyses to the laser-induced pain data in the literature. In both averaging and single trials, we have demonstrated that the pain-evoked event potentials can be extracted and further analyzed with careful design of statistical procedure, and that the ICA/BSS approaches show a promising role in analyzing the multichannel ECoG data recorded from the awake human subjects. Our results here have also validated our previous findings in the early investigations and the reported neurophysiological observations in the literature. This is encouraging in that it justifies the merits of blind signal processing for neurobiological or physiological data analysis. The next challenge of this line of research is to extract consistently less-dominant (in terms of power) and potentially important pain-related components that are beyond the LEPs from single trials, which will be the subject of future study.

We have focused on one particular type of blind signal processing tool (namely, ICA) in this paper. However, we make no claim that the choice is unique or optimal. Indeed, we have been aware of the strengthes and weaknesses of the ICA during the experimental investigations (e.g., Makeig et al. [25]), although other improved ICA models, such as the spatially constrained ICA (Ille et al. [35], Hesse and James [36]) or the temporally constrained ICA (James and Gibson [37]), can be considered. It is also noteworthy to point out several other powerful blind signal processing tools and statistical algorithms, whichmight be valuable for the future investigation:

- nonnegative matrix factorization (NMF) (e.g., Lee and Seung [38]), which is an approximate matrix factorization method for nonnegative data (e.g., spectra, or time-frequency map). Unlike ICA, the independence assumption is relaxed or unnecessary in NMF, on the other hand, extra constraints (such as the smoothness or sparsity) can be imposed for this statistical model.^12^
- parallel factor analysis (PARAFAC) (e.g., Bro [39]), which is a well-suited method for analyzing highdimensional tensorial data; PARAFAC can be viewed as a generalization of higher-order FA or highdimensional NMF (if additional nonnegativity constraint is imposed).
- common spatial subspace decomposition (CSSD) (Wang et al. [21]), which is a spatial filtering method for extracting signal components specific to one condition from multichannel electrode recordings given multiple task conditions. This kind of common spatial pattern algorithm may be used for evaluating the ECoG recordings under different task conditions; however, unlike the ICA method, it is a supervised algorithm that uses labeled data for classification.

In addition to the above-mentioned statistical tools, it would be also interesting to investigate the instantaneous brain activities and dynamics (Makeig et al. [25]), which may provide useful information of interactions inside the brain for specific patients with ECoG recordings. Finally, we believe what we reported here is only the first step towards a complete “statistical” understanding of the pain-evoked ECoG data, the substantiation of our observations, claims, and conclusionmade in this article would require more experimental verification of ECoG recordings in the future.

## Figures and Tables

**Table 1:** Summary of the experimental recordings of two human subjects.

Subject	Condition	Laser intensity	No. of electrodes	No. of runs	No. of trials at each run
1	Attention	720 mJ	89	3	38, 38, 38
1	Distraction	720 mJ	89	3	38, 38, 38

2	Attention	720 mJ	80	2	38, 38
2	Distraction	720 mJ	80	2	38, 38

2	Intensity	480 mJ	80	4	8, 12, 10, 10
2	Intensity	640 mJ	80	4	11, 11, 12, 10
2	Intensity	800 mJ	80	4	10, 10, 10, 10

**Table 2:** The relative power comparisons between prestimulus period (100 milliseconds) and poststimulus period (500 milliseconds) in the averaging-trials. The statistics are averaged over the total number of channels (namely, divided by 89 and 80 for subjects 1 and 2, resp.) and the relative time period. The values are unitless, reflecting the ratio among the normalized energy of the time-frequency map.

	Subject 1	Subject 1	Subject 2	Subject 2
	(counting)	(reading)	(counting)	(reading)
Ave. prestimulus total power	0.0667	0.0796	0.0748	0.0969
Ave. prestimulus *θ* power	0.0160	0.0265	0.0175	0.0333
Ave. prestimulus α power	0.0048	0.0105	0.0112	0.0102
Ave. prestimulus *β* power	0.0047	0.0078	0.0136	0.0087
Ave. prestimulus *γ* power	0.0021	0.0019	0.0026	0.0025

Ave. poststimulus total power	0.1867	0.1841	0.1850	0.1806
Ave. poststimulus *θ* power	0.0486	0.0617	0.0539	0.0502
Ave. poststimulus α power	0.0074	0.0124	0.0154	0.0160
Ave. poststimulus *β* power	0.0051	0.0073	0.0134	0.0104
Ave. poststimulus *γ* power	0.0024	0.0024	0.0033	0.0023

**Table 3:** Statistical hypothesis testing statistics of various extracted LEPs in averaging trials for subject 1. The Mann-Whitney *U*-test was applied to the “absolute value” of the raw samples, and the ANOVA *F*-test was applied to the logarithm transformation of the absolute value of the raw samples. The N/A implies that the samples are neither normally nor log-normally distributed and therefore cannot be used for ANOVA.

*P*-value	Counting	Counting	Reading	Reading
	(N150)	(P200)	(N150)	(P200)
*U*-test	.0029	.0013	7 × 10^−5^	.0269
*F*-test	.0003	N/A	6 × 10^−5^	.0183

**Table 4:** A comparison of main operations between the averaging and single-trial analyses.

Routine	Averaging trials	Single trials	Purpose
Averaging	Yes	No	Smoothing
FA+PCA	Yes	Yes	Noise and dimensionality reduction
first-stage ICA	Yes	Yes	Extracting independent sources
second-stage ICA	No	Optional	Fine tuning of the sources
Source identification	Yes	Yes	Locating the LEPs of interest
WVD	Optional	Optional	Visualization
Wavelet transform	Yes	Optional	*Z*-score transform
Statistical test	Optional	Optional	Testing hypothesis

**Table 5:** Comparative statistics of the relative power of the normalized wavelet scalogram followed by *Z*-score transformation (for subject 2, no. 14 electrode) in single-trial analysis. The mean and standard deviation (mean ±SD) statistics are calculated by averaging the number of trials in each run.

Run	No. of trials	Intensity	*θ* power	*α* power	*β* power	*γ* power
1a	8	480 mJ	0.10 ± 0.76	0.24 ± 0.76	0.33 ± 0.70	0.27 ± 0.62
1a	11	640 mJ	0.22 ± 0.83	0.09 ± 0.48	0.03 ± 0.30	0.10 ± 0.49
1a	10	800 mJ	0.57 ± 2.92	0.09 ± 0.72	−0.03 ± 0.39	0.14 ± 0.67

1b	12	480 mJ	0.38 ± 1.63	0.19 ± 0.73	0.25 ± 0.44	0.22 ± 0.40
1b	11	640 mJ	0.58 ± 0.91	0.22 ± 0.57	0.08 ± 0.58	0.05 ± 0.48
1b	10	800 mJ	0.66 ± 1.23	0.59 ± 0.79	0.47 ± 1.39	0.41 ± 0.92

2a	10	480 mJ	0.29 ± 0.61	0.40 ± 0.57	0.53 ± 1.26	0.43 ± 0.66
2a	12	640 mJ	0.76 ± 1.62	0.35 ± 0.93	0.21 ± 0.66	0.11 ± 0.61
2a	10	800 mJ	0.77 ± 2.11	0.24 ± 0.69	0.12 ± 0.33	0.36 ± 0.51

2b	10	480 mJ	−0.16 ± 0.30	−0.04 ± 0.31	0.17 ± 0.28	0.31 ± 0.38
2b	10	640 mJ	0.30 ± 0.93	0.34 ± 0.85	0.28 ± 0.84	0.05 ± 0.44
2b	10	800 mJ	0.64 ± 1.56	0.21 ± 0.59	0.10 ± 0.27	0.20 ± 0.33

**Table 6:** Comparative results of the estimated amplitudes and latencies of the LEPs (subject 2, under rating condition) from single and averaging trials. The last row indicate the selected number of single trials (by excluding some bad trials) used to evaluate the latencies.

	N2 (SI region)			P2 (SI region)		
Intensity (mJ)	480	640	800	480	640	800

Latency (milliseconds)	260	178	122	300	248	171
Amplitude (*μ*V)	−121 ± 18	−125 ± 23	−150 ± 41	112 ± 31	126 ± 55	98 ± 20
No. of trials	28	36	34	22	26	26

## Footnotes

^1^This is in contrast to the earliest component N1, which is a lateralized, relatively small negative peak.

^2^In the previous studies, the authors were not sure if the ECoG-LEPs correspond to the scalp-recorded LEPs (N2 and P2), therefore they used the nomenclatures N2* and P2**. Here, for simplicity, we use N2 and P2 for referring to these two LEPs. The LEPs can also be labeled by polarity and latency; when the latency is known, we also use N150 or P200 for the same reference purpose.

^3^The Matlab code is available at http://www-sigproc.eng.cam.ac.uk/~cf269.

^4^If only one-stage ICA is used, then **W** ^(1)^ = **I**, **y** ^(1)^ = **z**, and **W** = **W** ^(2)^.

^5^If the demixing matrix is square, then the matrix pseudoinverse **W** ^†^ will reduce to the matrix inverse **W** ^−1^.

^6^Generally, the greater the width parameter is, the better frequency resolution we can obtain; nevertheless, this is at the cost of sacrificing temporal resolution. The temporal resolution has a reciprocal relationship with respect to the frequency resolution.

^7^Specifically, the two LEP components are more significant and relatively robust in that they remain approximately unchanged when we vary (increase or decrease) the selected number of components by 1 or 2.

^8^This is in contrast to the two-stage ICA procedure in single-trials; this is often witnessed by the observation that the outcomes of the second-stage ICA are not much different from the results of the first-stage ICA

^9^It should be noteworthy that it is also possible to apply the logarithm transformation to the samples before theMann-Whitney test; in this case, the *P*-values will remain unchanged, except that the standard deviation will become smaller after the logarithm transformation.

^10^We call it surrogate single-trial LEP because the LEP is not estimated directly from individual single trial alone; instead, its recovery arises from the unmixing matrix that is estimated based on averaging all single trials.

^11^Typically, the window of temporal axis is centered at 150 milliseconds (or 200 milliseconds) with width 30 milliseconds, and the window of frequency axis covers from 4.5 to 6.5 Hz (with the resolution of 0.25 Hz for each frequency bin).

^12^For various implementation, see online resources and MATLAB toolboxes developed in our lab: http://www.bsp.brain.riken.jp/ICALAB/nmflab.html.

## APPENDIX

### MAXIMUM LIKELIHOOD ESTIMATION OF FACTOR ANALYSIS

Let us consider a general factor analysis(FA) model as follows:

(A.1) $$x_{t}=\mu +Bz_{t}+\varepsilon_{t},$$

where **x** *_t_* ∈ ℝ^*m*^ denotes the observed variable, *μ* denotes the mean vector, **z** *_t_* ∈ ℝ^*n*^ denotes the hidden variable called “factor,” and **B** is an *m* × *n* “loading matrix.” With the probabilistic assumptions that **z** *_t_* ∼ *𝒩* (**0**,**I**), ε*_t_* ∼ *𝒩* (**0**, Σ), and 𝔼[**z** *_t_ε_t_*] = **0**, then we may derive that

(A.2) $$\begin{array}{c} 𝔼[x_{t}]=\mu ,𝔼[x_{t}|z_{t}]=\mu +Bz_{t},\\ \text{Var}[x_{t}]=BB^{T}+\Sigma ,\text{Cov}[x_{t},z_{t}]=B. \end{array}$$

Let *θ* = (*μ*, **B**, Σ) denote the unknown parameters, then the log likelihood of the FA model is written as

(A.3) $$\begin{array}{ll} ℒ\left(\theta \right) & =\sum_{t=1}^{T}\text{In}\;p\left(x_{t}|\theta \right)\\ & =-\frac{T}{2}\text{In}\left|\Sigma \right|-\frac{1}{2}\sum_{t=1}^{T}x_{t}^{T}x_{t}\\ & -\frac{1}{2}\sum_{t=1}^{T}\left\{{\left(x_{t}-Bz_{t}\right)}^{T}\Sigma^{-1}\left(x_{t}-Bz_{t}\right)\right\}\\ & =-\frac{T}{2}\text{In}\left|\Sigma \right|-\frac{1}{2}\sum_{t=1}^{T}\text{tr}\left[x_{t}x_{t}^{T}\right]\\ & -\frac{1}{2}\sum_{t=1}^{T}\text{tr}\left[\left(x_{t}-Bz_{t}\right){\left(x_{t}-Bz_{t}\right)}^{T}\Sigma^{-1}\right], \end{array}$$

where tr[·] denotes the trace operator, and |Σ| denotes the determinant of **Σ**. Maximizing *ℒ*(*θ*) with respect to the unknown parameters yields the maximum likelihood estimate. An elegant solution can be obtained by using the iterative EM algorithm.

## References

[1] Bromm B, Lorenz J (1998). Neurophysiological evaluation of pain. *Electroencephalography and Clinical Neurophysiology*.

[2] García-Larrea L, Convers P, Magnin M (2002). Laser-evoked potential abnormalities in central pain patients: the influence of spontaneous and provoked pain. *Brain*.

[3] García-Larrea L, Peyron R, Laurent B, Mauguière F (1997). Association and dissociation between laser-evoked potentials and pain perception. *NeuroReport*.

[4] Tarkka IM, Treede R-D (1993). Equivalent electrical source analysis of pain-related somatosensory evoked potentials elicited by a CO_2_ laser. *Journal of Clinical Neurophysiology*.

[5] Iannetti GD, Niazy RK, Wise RG (2005). Simultaneous recording of laser-evoked brain potentials and continuous, high-field functional magnetic resonance imaging in humans. *NeuroImage*.

[6] Lenz FA, Rios M, Zirh A, Chau D, Krauss G, Lesser RP (1998). Painful stimuli evoke potentials recorded over the human anterior cingulate gyrus. *Journal of Neurophysiology*.

[7] Ohara S, Crone NE, Weiss N, Lenz FA (2004). Attention to a painful cutaneous laser stimulus modulates electrocorticographic event-related desynchronization in humans. *Clinical Neurophysiology*.

[8] Ohara S, Crone NE, Weiss N, Treede R-D, Lenz FA (2004). Cutaneous painful laser stimuli evoke responses recorded directly from primary somatosensory cortex in awake humans. *Journal of Neurophysiology*.

[9] Ohara S, Crone NE, Weiss N, Vogel H, Treede R-D, Lenz FA (2004). Attention to pain is processed at multiple cortical sites in man. *Experimental Brain Research*.

[10] Legrain V, Guérit J-M, Bruyer R, Plaghki L (2002). Attentional modulation of the nociceptive processing into the human brain: selective spatial attention, probability of stimulus occurrence, and target detection effects on laser evoked potentials. *Pain*.

[11] Legrain V, Bruyer R, Guérit J-M, Plaghki L (2003). Nociceptive processing in the human brain of infrequent task-relevant and task-irrelevant noxious stimuli. A study with event-related potentials evoked by CO_2_ laser radiant heat stimuli. *Pain*.

[12] Mouraux A, Guérit JM, Plaghki L (2003). Non-phase locked electroencephalogram (EEG) responses to CO_2_ laser skin stimulations may reflect central interactions between A*∂*- and C-fibre afferent volleys. *Clinical Neurophysiology*.

[13] Ploner M, Gross J, Timmermann L, Pollok B, Schnitzler A (2006). Pain suppresses spontaneous brain rhythms. *Cerebral Cortex*.

[14] Lee T-W, Girolami M, Sejnowski TJ (1999). Independent component analysis using an extended infomax algorithm for mixed subgaussian and supergaussian sources. *Neural Computation*.

[15] Cao J, Murata N, Amari S-I, Cichocki A, Takeda T (2002). Independent component analysis for unaveraged single-trial MEG data decomposition and single-dipole source localization. *Neurocomputing*.

[16] Cao J, Murata N, Amari S-I, Cichocki A, Takeda T (2003). A robust approach to independent component analysis of signals with high-level noise measurements. *IEEE Transactions on Neural Networks*.

[17] Makeig S, Westerfield M, Jung T-P (2002). Dynamic brain sources of visual evoked responses. *Science*.

[18] Anemüller J, Sejnowski TJ, Makeig S (2003). Complex independent component analysis of frequency-domain electroencephalographic data. *Neural Networks*.

[19] Miwakeichi F, Martínez-Montes E, Valdés-Sosa PA, Nishiyama N, Mizuhara H, Yamaguchi Y (2004). Decomposing EEG data into space-time-frequency components using parallel factor analysis. *NeuroImage*.

[20] Browne M, Cutmore TRH (2002). Low-probability event-detection and separation via statistical wavelet thresholding: an application to psychophysiological denoising. *Clinical Neurophysiology*.

[21] Wang Y, Berg P, Scherg M (1999). Common spatial subspace decomposition applied to analysis of brain responses under multiple task conditions: a simulation study. *Clinical Neurophysiology*.

[22] Galka A, Yamashita O, Ozaki T, Biscay R, Valdés-Sosa P (2004). A solution to the dynamical inverse problem of EEG generation using spatiotemporal Kalman filtering. *NeuroImage*.

[23] Cichocki A (2004). Blind signal processing methods for analyzing multichannel brain signals. *International Journal of Bioelectromagtism*.

[24] Cichocki A, Wenger MJ, Schuster C (2006). Generalized component analysis and blind source separation methods for analyzing multichannel brain signals. *Statistical and Process Models of Cognitive Aging*.

[25] Makeig S, Debener S, Onton J, Delorme A (2004). Mining event-related brain dynamics. *Trends in Cognitive Sciences*.

[26] Mørup M, Hansen LK, Herrmann CS, Parnas J, Arnfred SM (2006). Parallel factor analysis as an exploratory tool for wavelet transformed event-related EEG. *NeuroImage*.

[27] Cichocki A, Amari S (2002). *Adaptive Blind Signal and Image Processing*.

[28] Févotte C, Doncarli C (2004). Two contributions to blind source separation using time-frequency distributions. *IEEE Signal Processing Letters*.

[29] Mallat S (1999). *A Wavelet Tour of Signal Processing*.

[30] Tallon-Baudry C, Bertrand O, Delpuech C, Pernier J (1996). Stimulus specificity of phase-locked and non-phase-locked 40 Hz visual responses in human. *The Journal of Neuroscience*.

[31] Düzel E, Habib R, Schott B (2003). A multivariate, spatiotemporal analysis of electromagnetic time-frequency data of recognition memory. *NeuroImage*.

[32] Delorme A, Makeig S (2004). EEGLAB: an open source toolbox for analysis of single-trial EEG dynamics including independent component analysis. *Journal of Neuroscience Methods*.

[33] Delorme A, Makeig S, Fabre-Thorpe M, Sejnowski T (2002). From single-trial EEG to brain area dynamics. *Neurocomputing*.

[34] Ohara S, Crone NE, Weiss N, Treede R-D, Lenz FA (2004). Amplitudes of laser evoked potential recorded from primary somatosensory, parasylvian and medial frontal cortex are graded with stimulus intensity. *Pain*.

[35] Ille N, Beucker R, Scherg M (2001). Spatially constrained independent component analysis for artifact correction in EEG and MEG. *NeuroImage*.

[36] Hesse CW, James CJ (2005). The FastICA algorithm with spatial constraints. *IEEE Signal Processing Letters*.

[37] James CJ, Gibson OJ (2003). Temporally constrained ICA: an application to artifact rejection in electromagnetic brain signal analysis. *IEEE Transactions on Biomedical Engineering*.

[38] Lee DD, Seung HS (1999). Learning the parts of objects by non-negative matrix factorization. *Nature*.

[39] Bro R (1998). *Multi-way analysis in the food industry: models, algorithms and applications, Ph.D. thesis*.

